# Successful treatment of lichen planopilaris with topical cyclosporine: A case series

**DOI:** 10.1016/j.jdcr.2025.02.011

**Published:** 2025-03-10

**Authors:** Alireza Jafarzadeh, Nazila Salami, Afsaneh Sadeghzadeh Bazargan, Mohammadreza Ghassemi, Sepideh Salehi, Azadeh Goodarzi

**Affiliations:** a Department of Dermatology, Rasool Akram Medical Complex Clinical Research Development Center (RCRDC), School of Medicine, Iran University of Medical Sciences (IUMS), Tehran, Iran; b Isfahan University of Medical Science, Dental School, Tehran, Iran

**Keywords:** alopecia, cyclosporine, FFA, lichen planopilaris, LPP, topical cyclosporine

## Introduction

Lichen planus is an inflammatory disease affecting the skin and mucous membranes, classified into several types, one of which is lichen planopilaris (LPP).^1^ LPP is a primary cicatricial alopecia characterized by lymphocytic infundibulo-isthmic inflammation, leading to the destruction of follicular bulge stem cells.^2^^,^^3^ Common symptoms and signs include increased hair shedding, itching, scaling, burning, and tenderness. Differentiation from other forms of cicatricial alopecia can be achieved through a thorough evaluation of clinical, histopathologic, and immunohistopathologic findings.^4^

Several treatment options have been suggested for LPP, including mycophenolate mofetil, hydroxychloroquine, methotrexate, tofacitinib, pioglitazone, and oral cyclosporine, among others. Each treatment is associated with varying degrees of recovery, relapse, and side effects.^5^

Cyclosporine-A (Cyc-A) was originally used as a systemic treatment for patients undergoing solid organ transplants and those with graft-versus-host disease. Topical Cyc-A is particularly advantageous for treating mucocutaneous conditions due to its reduced systemic side effects. It has been found effective for conditions such as oral lichen planus, psoriasis, burning mouth syndrome, pyoderma gangrenosum, and Zoon's balanitis.^6^^,^^7^

Considering the increasing prevalence of LPP and its potential effects on both appearance and psychological well-being, along with the necessity for long-term use of available treatments and their related side effects—especially in patients who are unable to undergo systemic treatment for various reasons—this study was designed. This case series reports the successful treatment of LPP with topical cyclosporine. Additionally, a comprehensive review of the existing literature on prior studies has been conducted.

## Case report

### Patient demographics

Seven patients were selected from those referred to the dermatology clinic, with an average age of 42.4 years (±9.25 y standard deviation), and a 95% confidence interval of (35.56, 49.24). All patients included in the study were women (Table I).

**Table I:** Characteristics of patients with lichen planopilaris treated with topical cyclosporine

Patients	Sex	Age	Time from diagnosis to initiation of treatment	Previous treatments	Medical history and comorbidities
1	Female	35	3 mo	Clobetasol Propionate Lotion 0.05%, to be applied daily.	-
2	Female	36	1 mo	-	Hyperlipidemia.
3	Female	20	6 mo	Mycophenolate mofetil 2 g daily	-
4	Female	49	3 mos	-	-
5	Female	57	1 y	Clobetasol Propionate Lotion 0.05%, to be applied daily, and Hydroxychloroquine 200 mg, to be taken twice daily.	Hypertension
6	Female	57	1 mo	-	Hypertension and diabetes mellitus.
7	Female	43	2 wk	-	-

### Clinical presentation

#### Diagnosis

Each patient was diagnosed with LPP through clinical evaluation, and the diagnosis was confirmed by biopsy. The duration between the confirmation of the diagnosis and the initiation of therapy among the 7 patients varied from 2 weeks to 1 year, with an average of 3.79 months (±3.32 months standard deviation). Three patients (42.86%) had a history of previous treatments, while 4 patients opted not to receive treatment for various reasons prior to our visit. Previous treatments included Clobetasol Propionate Lotion 0.05% (to be applied daily), mycophenolate mofetil (2 g daily), and hydroxychloroquine (200 mg, to be taken twice daily). Associated comorbidities were reported in 3 patients (42.86%), specifically hyperlipidemia, hypertension, and diabetes mellitus. At the time of our visit, all comorbidities were well-controlled (Table I).

#### Symptoms

The patients presented with a range of symptoms, including pain in 3 patients (42.9%), itching in all patients (100%), hair loss in all patients (100%), scalp erythema in all patients (100%), and perifollicular erythema in 5 patients (71.4%). Additionally, 2 patients (28.6%) exhibited all symptoms: pain, itching, hair loss, scalp erythema, and perifollicular erythema.

### Treatment protocol

All patients were treated with a topical cyclosporine formulation comprised of 250 mg of cyclosporine, 80 cc of propylene glycol, and 15 cc of distilled water. The treatment was applied nightly to the affected scalp area at a dosage of 1 cc for a duration of 6 months.

### Follow-up assessments

Follow-up evaluations were conducted at intervals of 1 month, 3 months, 5 months, and 6 months after initiating treatment to monitor progress and assess treatment efficacy.

### Outcome measures

#### Lichen Planopilaris Activity Index (LPPAI)

**Fig 1:**
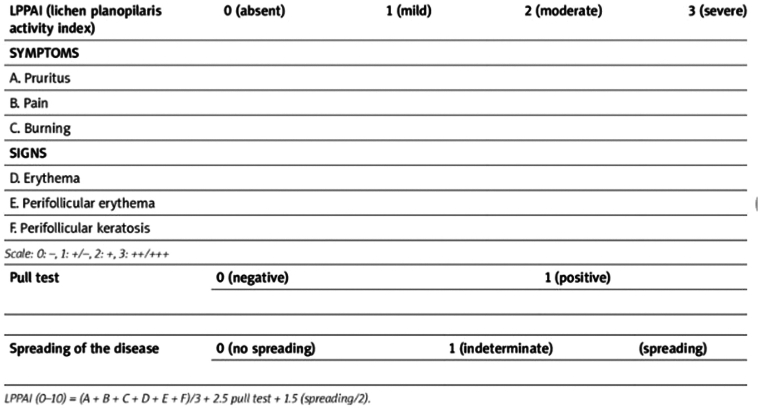
Lichen Planopilaris Activity Index (LPPAI).

Disease severity was assessed using the LPPAI^8^, ^9^ (Fig 1), a composite scale encompassing both objective and subjective criteria, with scores ranging from 0 to 10. Notably, 3 patients achieved a score of 0 by the end of the 6-month observation period. Initially, the average LPPAI score at the first visit stood at 6.92, then progressively decreased to 5.17 after 1 month, 3.62 after 3 months, 1.84 at 5 months, and ultimately reached 1.39 by the sixth month (Table II). The standard deviation for the LPPAI values was approximately ±2.03.

**Table II:** Changes in LPP Activity Index during follow-up

Patients	Initial visit	1 mo post-treatment	3 mo post-treatment	5 mo post-treatment	6 mo post-treatment
1	6.83	3	1.66	0.33	0
2	8.33	7.33	7	5.24	5.24
3	7.79	7	3.16	1.74	1.03
4	6.6	5.99	5.3	1.08	0
5	6.58	4.25	3.41	3.5	2.83
6	7.24	5.83	4.5	0.99	0.66
7	5.1	2.83	0.33	0	0

#### Pull test results

Pull tests were negative for all patients except for one individual (Patient 2), indicating a positive response to treatment.

#### Symptom assessment

**Fig 2:**
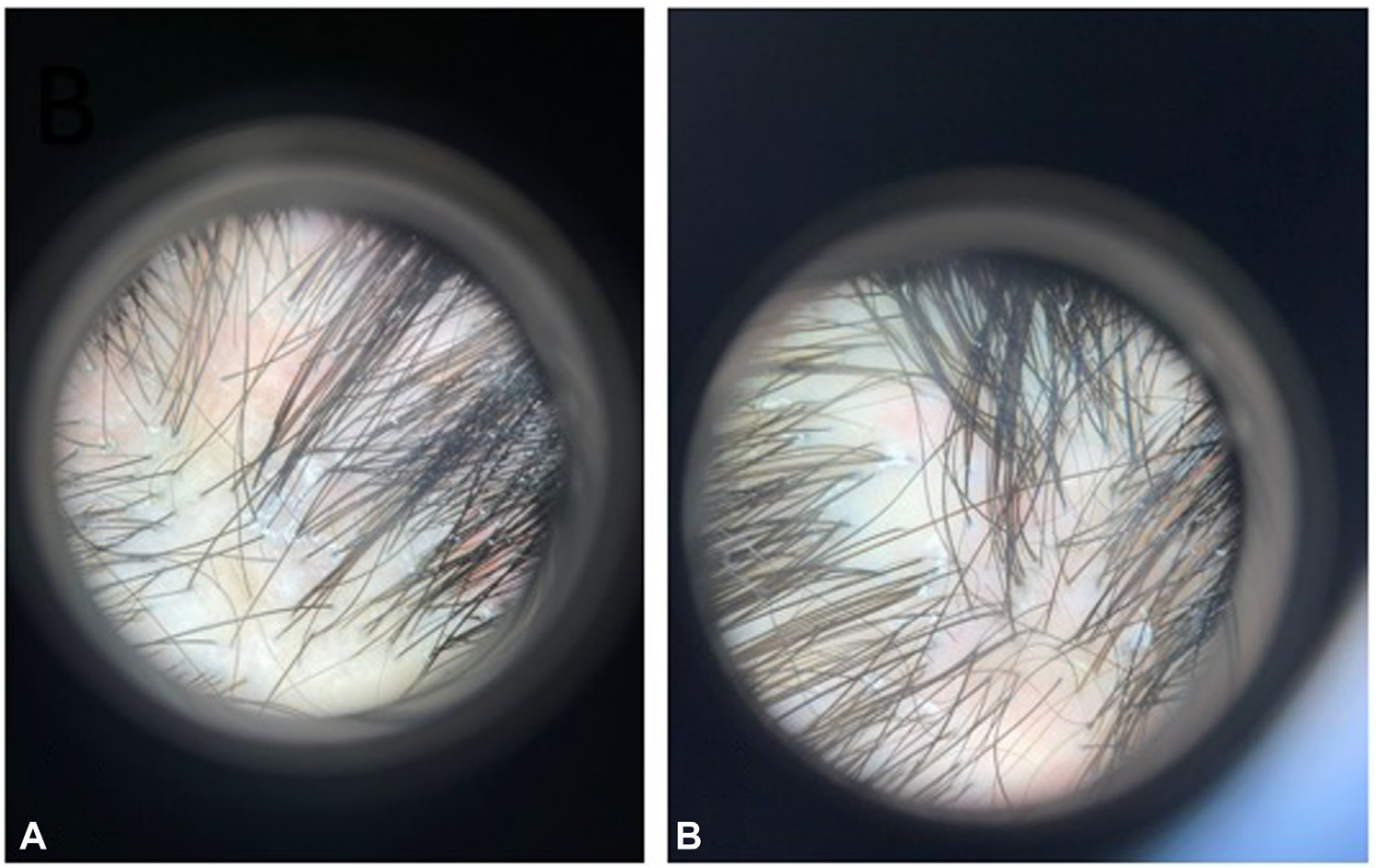
Clinical recovery of LPP lesions 6 months post-treatment. **A,** Before treatment; (**B**): 6 months post-treatment.

**Fig 3:**
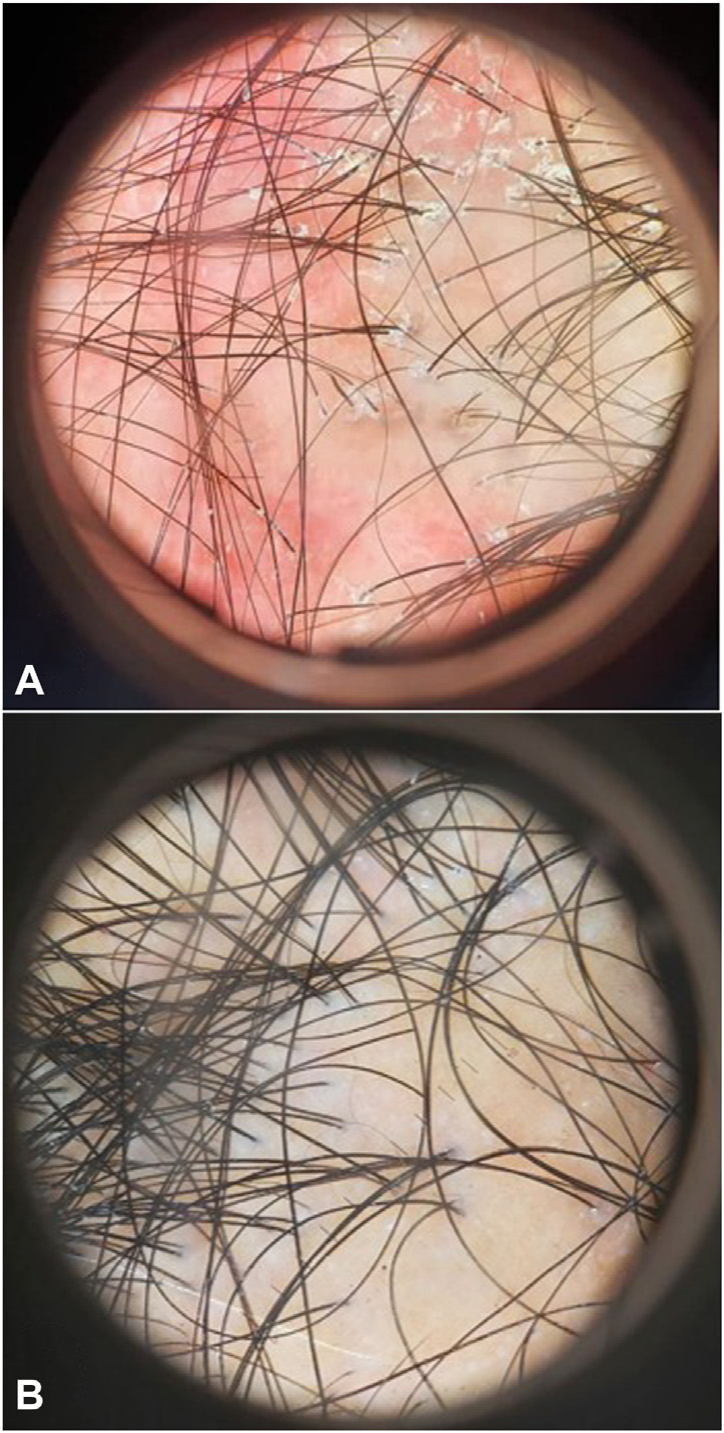
Clinical improvement of erythema and scale 6 months post-treatment. **A,** Before treatment; (**B**): 6 months post-treatment.

Throughout the 6-month treatment duration, a noteworthy decline was observed in scalp erythema, itching, pain, perifollicular erythema, and disease progression (Figs 2 and 3). Despite these positive trends, it is important to note that 2 patients still indicated experiencing itching beyond the 6-month mark. Moreover, although no patients specifically noted pain, one individual reported an enduring burning sensation. Notably, 2 patients displayed persistent scalp erythema, albeit with reduced intensity, and 2 others exhibited a decrease in perifollicular scalp erythema (Table II).

#### Patient satisfaction

Throughout the treatment sessions, an increasing number of patients expressed satisfaction with the therapeutic outcomes. However, by the end of the treatment period, 3 patients remained dissatisfied with the results (see Table III).

**Table III:** Patients' satisfaction during follow-up

Patients	Initial visit	1 mo post-treatment	3 mo post-treatment	5 mo post-treatment	6 mo post-treatment
1	-	Great	Great	Excellent	Excellent
2	-	Without satisfaction	Low	Low	Without satisfaction
3	-	Without satisfaction	Middle	Low	Without satisfaction
4	-	Great	Great	Excellent	Excellent
5	-	Middle	Low	Low	Without satisfaction
6	-	Middle	Great	Great	Great
7	-	Middle	Great	Excellent	Excellent

#### Tolerability and side effects

Overall, treatment tolerability was excellent among all patients, with none reporting adverse side effects from the topical cyclosporine application.

## Discussion

LPP is a type of scarring alopecia that predominantly affects middle-aged women, with an estimated incidence ranging from 1% to 7%. Patients often experience thinning hair, which may be associated with itching or tenderness of the scalp. In the early stages, actively inflamed lesions are characterized by perifollicular erythema and hyperkeratosis.^9^^,^^10^

Timely diagnosis and early treatment are crucial due to the scarring nature of alopecia associated with this condition. Currently, therapeutic options include drugs such as hydroxychloroquine, methotrexate, cyclosporine, and mycophenolate mofetil.^11^^,^^12^ However, the use of these treatments presents challenges, including their variable effectiveness, the likelihood of recurrence, and potential side effects from long-term use.

The occurrence of side effects and the recurrence of lesions after discontinuing treatment have shifted attention toward topical therapies. In this context, the study by Lajevardi et al^13^ compared the effectiveness of topical clobetasol lotion (0.05% applied once nightly for 6 m) with oral pioglitazone in treating LPP. The results indicated that both methods effectively controlled the disease. In the clobetasol group, LPPAI scores at baseline and after treatment were 4.68 ± 1.97 and 2.59 ± 0.97, respectively. The treatment duration and application method for clobetasol in this study were similar to those for topical cyclosporine in our investigation. Remarkably, all 7 patients in our study exhibited a further decrease in LPPAI, with 3 patients achieving a score of zero.

Continuing the review of topical treatments for LPP, a study by Chen et al^14^ examined the effectiveness of topical tofacitinib 2% cream used either as monotherapy or as an adjunctive treatment for 9 months. The results showed a significant reduction LPPAI scores, with mean scores before and after treatment at 3.03 and 1.40, respectively (*P* < .0001). Additionally, there was an overall decrease of 48% in LPPAI scores at the 6-month mark. However, it's worth noting that the reduction in LPPAI scores observed in this study was less than that reported by all patients in our study.

The systematic review by Sadeghi et al^6^ suggests that topical Cyc-A is an effective treatment for various conditions, including oral lichen planus, psoriasis, burning mouth syndrome, pyoderma gangrenosum, and Zoon's balanitis. Some patients experienced side effects such as dysphagia, burning sensations, lip swelling, and gastrointestinal issues with Cyc-A mouthwash. In contrast, the lipogel formulation was associated with milder side effects, including erythema, dryness, and skin fissuring. Overall, topical Cyc-A is a promising alternative to conventional treatments for immune-mediated mucocutaneous disorders. Additionally, it is considered a safe and effective option for long-term use, as it minimizes the adverse effects commonly associated with prolonged steroid therapy.

Frontal fibrosing alopecia (FFA) shares many similarities with LPP in terms of pathogenesis. Both conditions can lead to scarring alopecia; however, FFA primarily affects the hairline and eyebrows. According to a study by Chen et al,^14^ topical tofacitinib has been found to be effective in improving both FFA and LPP.^14^

Treatment options for FFA and LPP are similar in many aspects and include topical therapies such as high-potency corticosteroids, intralesional corticosteroids, and calcineurin inhibitors. The main systemic treatments consist of 5-alpha-reductase inhibitors, hydroxychloroquine, and retinoids. Additionally, some studies have reported the use of tetracyclines, methotrexate, pioglitazone, naltrexone, and JAK inhibitors, such as tofacitinib.^15^

In summary, it is important to highlight that the topical cyclosporine formulation used in our study resulted in improvements in LPPAI scores. Notably, 3 patients achieved complete recovery by the end of the treatment period, demonstrating a considerably higher recovery rate compared to other topical treatments reported in previous studies.

## Conflicts of interest

None disclosed.

## References

[1] Bazargan A.S., Jafarzadeh A., Nobari N.N. (2023). Successful treatment of resistant plantar ulcerative lichen planus with tofacitinib: a case report and comprehensive review of the literature. Clin Case Rep.

[2] Jafari M.A., Bazgir G., Hosseini-Baharanchi F.S., Jafarzadeh A., Goodarzi A. (2024). Efficacy and safety of laser therapy and phototherapy in cicatricial and NonCicatricial alopecia: a systematic review study. Health Sci Rep.

[3] Sadeghzadeh Bazargan A., Tavana Z., Dehghani A. (2024). The efficacy of the combination of topical minoxidil and oral spironolactone compared with the combination of topical minoxidil and oral finasteride in women with androgenic alopecia, female and male hair loss patterns: a blinded randomized clinical trial. J Cosmet Dermatol.

[4] Kang H., Alzolibani A.A., Otberg N., Shapiro J. (2008). Lichen planopilaris. Dermatol Ther.

[5] Fatemi F., Esfahanian F., Asilian A., Mohaghegh F., Saber M. (2022). Comparative efficacy study combination of oral methotrexate and prednisolone versus oral methotrexate in patients with lichen planopilaris. Dermatol Res Pract.

[6] Sadeghi S., Kalantari Y., Seirafianpour F., Goodarzi A. (2022). A systematic review of the efficacy and safety of topical cyclosporine in dermatology. Dermatol Ther.

[7] Amber T., Tabassum S. (2020). Cyclosporin in dermatology: a practical compendium. Dermatol Ther.

[8] Chiang C., Sah D., Cho B.K., Ochoa B.E., Price V.H. (2010). Hydroxychloroquine and lichen planopilaris: efficacy and introduction of Lichen Planopilaris Activity Index scoring system. J Am Acad Dermatol.

[9] Xie F., Lehman J.S. (2022). Lichen planopilaris. Mayo Clin Proc.

[10] Jafarzadeh A., Pour Mohammad A., Keramati H., Zeinali R., Khosravi M., Goodarzi A. (2024). Regenerative medicine in the treatment of specific dermatologic disorders: a systematic review of randomized controlled clinical trials. Stem Cell Res Ther.

[11] Svigos K., Yin L., Fried L., Lo Sicco K., Shapiro J. (2021). A practical approach to the diagnosis and management of classic lichen planopilaris. Am J Clin Dermatol.

[12] Williams J., Lacy A., Ranpariya V.K., Pichardo R.O. (2023). Treatment patterns for hydroxychloroquine, methotrexate, and mycophenolate mofetil in lichen planopilaris: a retrospective chart review. J Cutan Med Surg.

[13] Lajevardi V., Ghiasi M., Balighi K. (2022). Efficacy and safety of oral pioglitazone in the management of lichen planopilaris in comparison with clobetasol: a randomized clinical trial. Dermatol Ther.

[14] Chen L.C., Ogbutor C., Kelley K.J., Senna M.M. (2024). Topical tofacitinib for patients with lichen planopilaris and/or frontal fibrosing alopecia. J Am Acad Dermatol.

[15] Fechine C.O.C., Valente N.Y.S., Romiti R. (2022). Lichen planopilaris and frontal fibrosing alopecia: review and update of diagnostic and therapeutic features. An Bras Dermatol.

